# A case of severe Mycoplasma pneumoniae pneumonia complicated by necrotizing tracheobronchitis with a review of the literature

**DOI:** 10.3389/fped.2026.1753605

**Published:** 2026-04-15

**Authors:** Chunyan Gao, Wujun Jiang, Yinfang Dai, Lina Xu, Meijuan Wang, Huiquan Sun

**Affiliations:** Department of Respiratory Medicine, Children's Hospital of Soochow University, Soochow, China

**Keywords:** bronchoscopy, children, Mycoplasma pneumoniae, necrotizing tracheobronchitis, severe Mycoplasma pneumoniae pneumonia

## Abstract

**Objective:**

To report a case of severe Mycoplasma pneumoniae pneumonia complicated with necrotizing laryngotracheobronchitis, and to detail its clinical manifestations, auxiliary examinations and treatment process, so as to enhance clinicians’ understanding of necrotizing laryngotracheobronchitis and emphasize the indispensable role of bronchoscopy in the diagnosis and treatment of necrotizing laryngotracheobronchitis.

**Methods:**

Report on a child with severe Mycoplasma pneumoniae pneumonia complicated by necrotizing laryngotracheobronchitis; the child was a 7 - year - old boy; the clinical features included recurrent high fever, cough and hoarseness; physical examination revealed fair spirits, pharyngeal congestion, grade I tonsillar enlargement, coarse breath sounds in both lungs, and no rales heard; auxiliary examinations confirmed Mycoplasma pneumoniae infection; under bronchoscopy, necrosis of the bronchial mucosa and exposure of cartilage were observed, and the pathological examination of the lesion showed inflammatory necrosis; after anti - infection treatment and multiple bronchoscopic lavage treatments, the child's condition improved and he was discharged from the hospital.

**Conclusion:**

Necrotizing laryngotracheobronchitis can present with diffuse necrosis of the airway mucosa, has an acute onset and a severe disease progression, and can lead to death; in this case, the child developed necrosis of the tracheal and bronchial mucosa after Mycoplasma pneumoniae infection, and there is currently no report of necrotizing laryngotracheobronchitis occurring after Mycoplasma pneumoniae infection; in treatment, anti - infection treatment should be combined with bronchoscopic lavage treatment.

Mycoplasma pneumoniae pneumonia (MPP) is the most common community-acquired pneumonia (CAP) in children over the age of 5 in China. Although MPP itypically manifests as a mild and self-limiting illness, certain children may progress to severe Mycoplasma pneumoniae pneumonia (SMPP). In 2023, there was a notable increase in the incidence of SMPP, possibly linked to a nationwide outbreak of MPP. Children with SMPP frequently experience complications both within and beyond the respiratory system, raising the likelihood of long-term sequelae. Recent advancements in bronchoscopy and pulmonary lavage have significantly enhanced the acute-phase treatment outcomes for SMPP in children, leading to a marked reduction in complications and sequelae. Although Mycoplasma pneumoniae (*M. pneumoniae)* infection can result in mucosal ulceration and necrosis, reports of necrotizing tracheobronchitis subsequent to such infections are scarce. Necrotizing tracheobronchitis represents a distinct form of bronchitis characterized by acute diffuse necrotizing inflammation of the laryngeal, tracheal, and bronchial mucosa, commonly associated with pathogens like Aspergillus and Staphylococcus aureus. This report presents a case of necrotizing tracheobronchitis following *M. pneumoniae* infection, showcasing a unique clinical scenario.

## Case data

### Present illness

Admitted on 2023-09-01, presenting a medical history of “8 days of fever and 6 days of cough.” The child initially developed a high fever 8 days prior, peaking at 40 °C, which temporarily subsided to normal levels upon administration of “ibuprofen.” However, the fever would return after approximately 3 h, without any accompanying chills or convulsions. Initially, the child presented with a sore throat but no cough. Subsequently, the child sought medical attention at a local hospital where a blood routine test revealed: white blood cell count of 9.5 × 10^9/L, with neutrophils accounting for 57%, CRP levels at 7 mg/L, and a positive result for *M. pneumoniae* IgM antibodies. The child received oral medication (details not specified), however, there was no observed improvement in the condition. Six days ago, the child began experiencing a cough, initially mild in nature, which progressively evolved into paroxysmal episodes producing sputum. This was accompanied by hoarseness, with no reported symptoms of shortness of breath, wheezing, or respiratory distress. Subsequently, the child revisited a local hospital where a chest x-ray revealed heightened and indistinct markings in both lungs. Treatment involving “azithromycin” and “methylprednisolone” was initiated for a day; however, no significant improvement was observed in the child's condition. From 2023-08-29 to 2023-08-31, the child was hospitalized at a local medical facility, where a chest CT scan revealed a sputum plug lodged in the bronchus of the anterior basal segment of the lower lobe of the left lung. Treatment involving “cefuroxime,” “erythromycin,” and “methylprednisolone” was administered; however, the child continued to experience fever and worsening cough, along with post-coughing nausea and hoarseness, although there were no reported breathing difficulties. Subsequently, the child was referred to our hospital for further evaluation and care. A subsequent chest CT scan exhibited minor inflammation in the lower lobe of the left lung, prompting admission to our facility for ongoing assessment and treatment. Throughout the illness, the child maintained an average level of alertness, experienced a decreased food intake by half, had normal sleep patterns, and regular bowel movements.

### Admission examination

Temperature 38 °C, pulse 95 beats per minute, respiration 20 breaths per minute, blood pressure 96/60 mmHg, weight 19.9 kg, oxygen saturation 98%. The child was conscious with an average spirit, exhibiting a soft neck with pharyngeal congestion and grade I tonsil enlargement. Auscultation revealed coarse breath sounds in both lungs without rales, alongside strong heart sounds with a regular rhythm and no murmurs. Upon abdominal examination, the abdomen was soft with no palpable masses, and the extremities were warm.

### Auxiliary examinations

**Figure 1:**
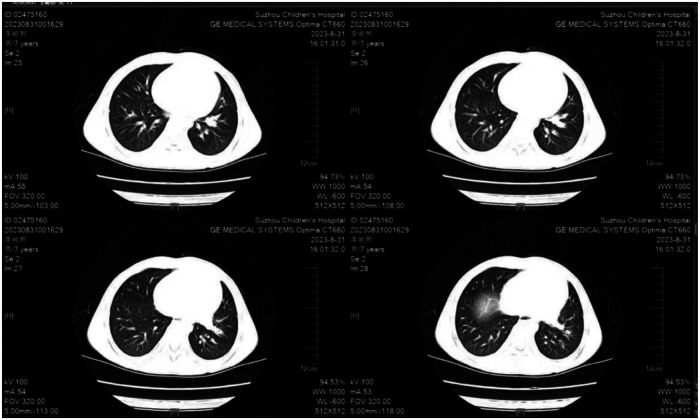
Chest CT (2023-8-31): increased bronchovascular markings in both lungs, with a few patchy opacities in the left lower lobe.

**Figure 2:**
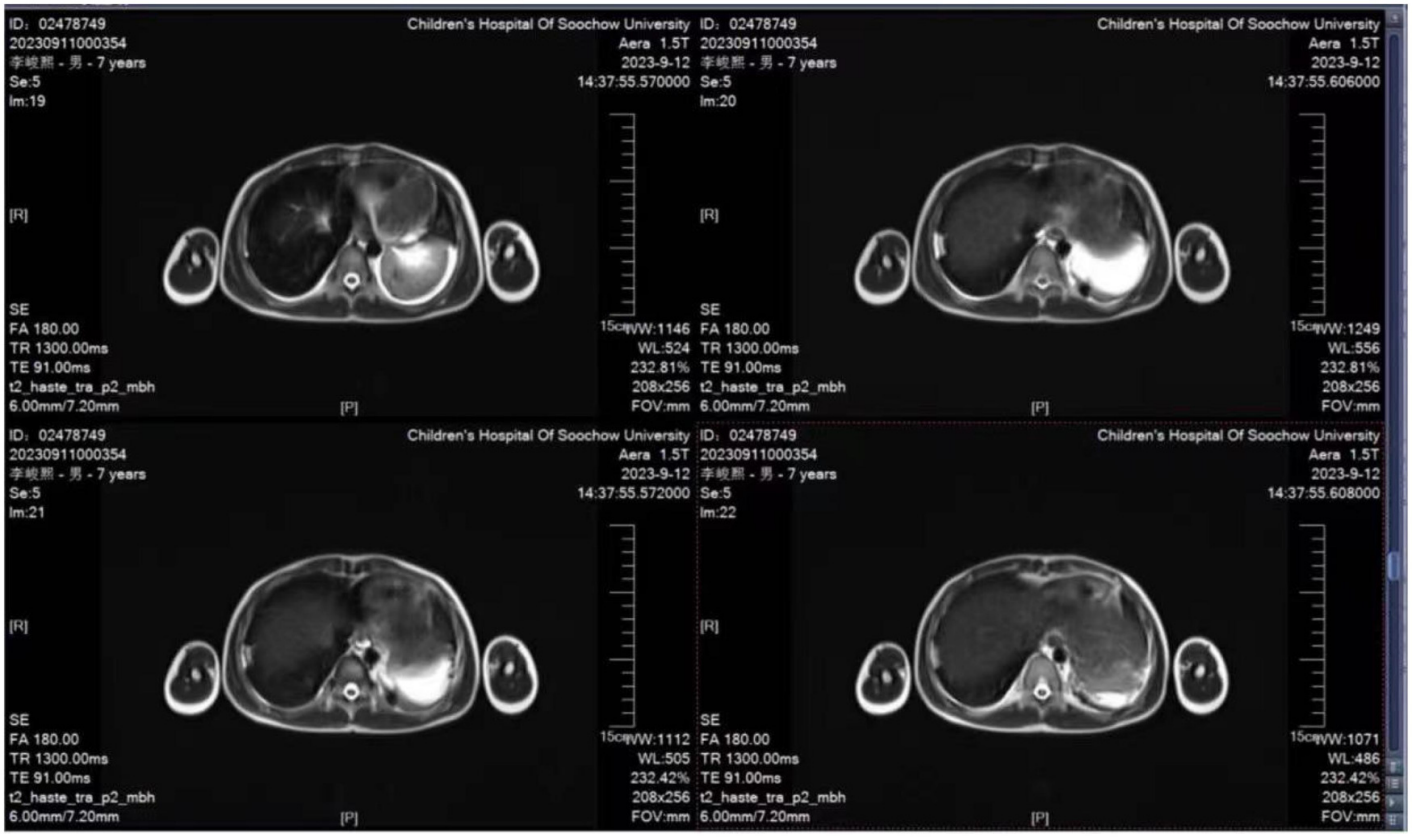
Chest MRI (2023-09-12) showed large pressure pressure hyperintensity in the left lower lung area and flocculate pressure hyperintensity in the right lobe of the lower lung.

**Figure 3:**
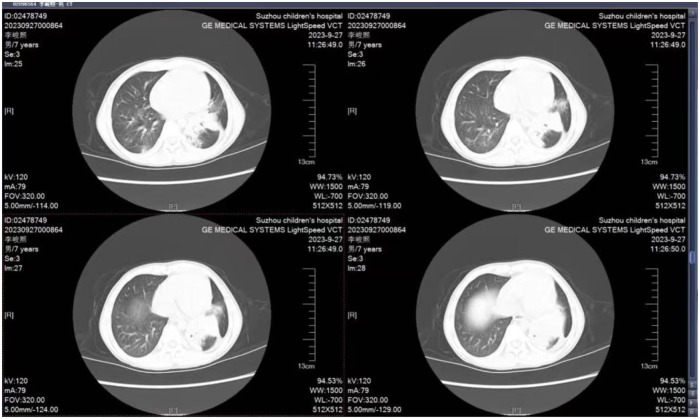
Chest CT (2023-09-27): slice and cable high-density shadows in both lungs, with the left lower lobe.

**Figure 4:**
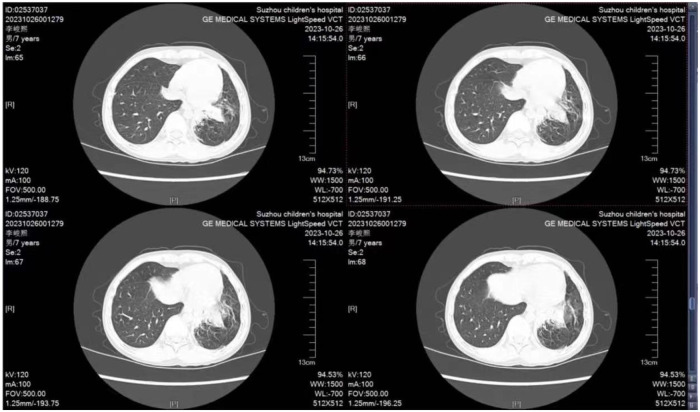
Chest high-resolution CT (2023-10-26) with visible slice and cable-like high-density shadows in both lungs, with the left lower lobe.

**Figure 5:**
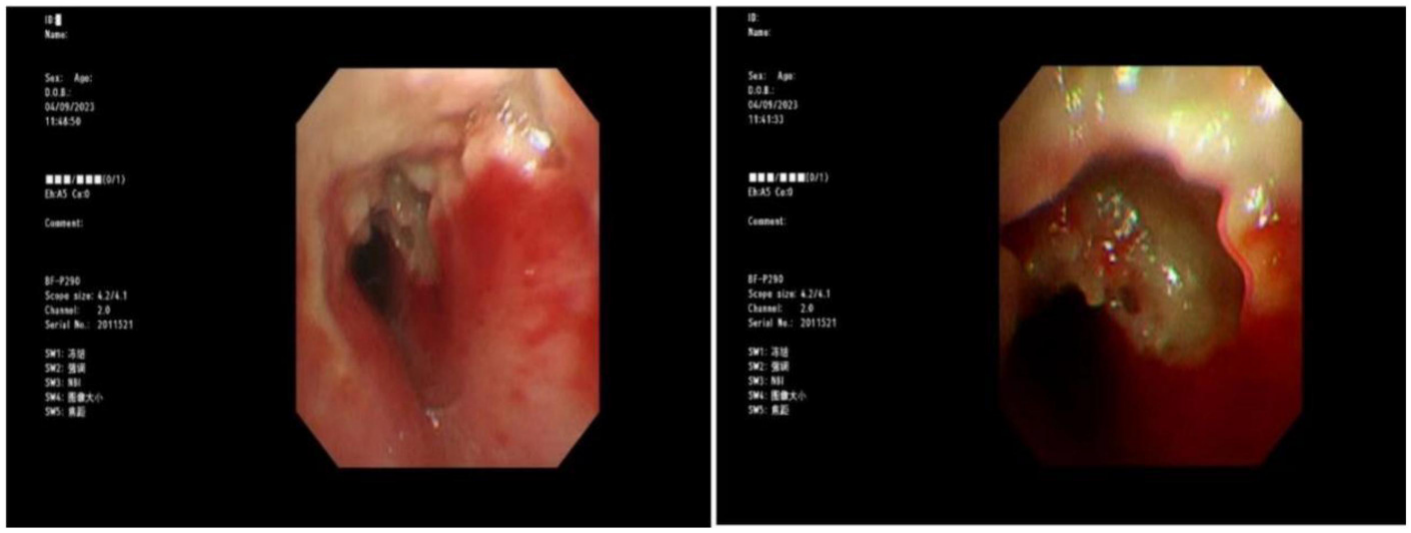
Bronchoscope (2023-09-04): bronchial mucosa erosion, visible exposed cartilage.

**Figure 6:**
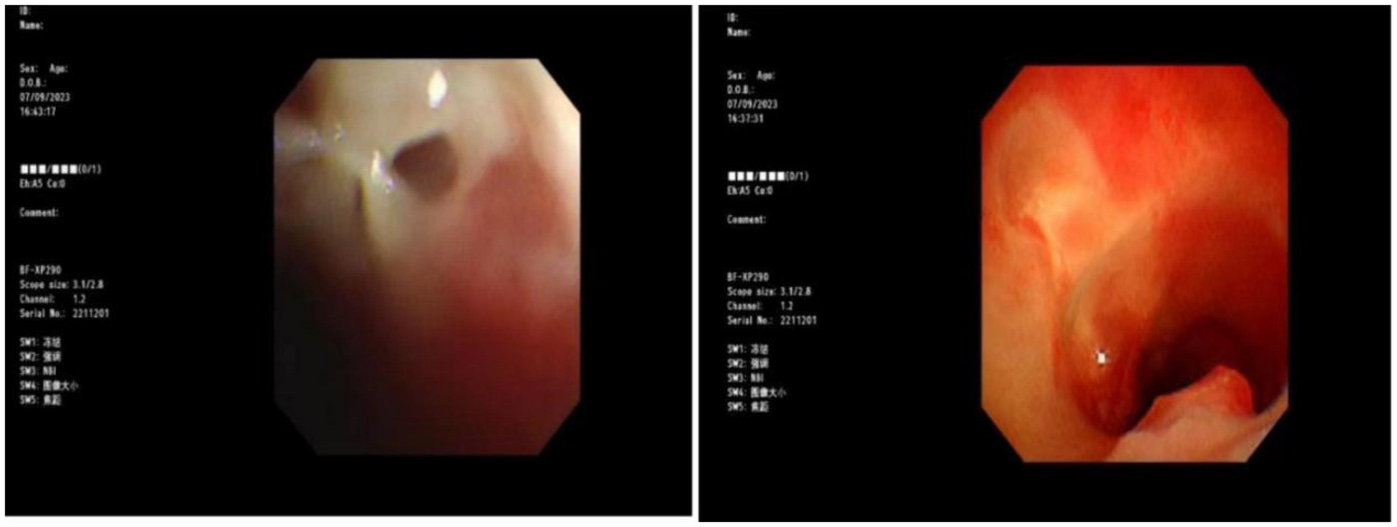
Bronchoscope (2023-09-07): the erosion of bronchial mucosa was improved and the cartilage was exposed.

**Figure 7:**
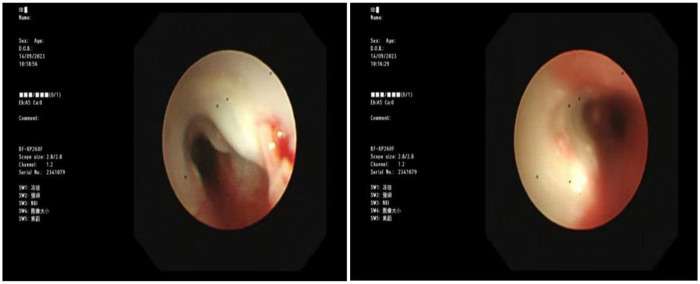
Bronchoscope (2023-09-14): the bronchial mucosa covered part of the cartilage, and the cartilage exposed was improved.

**Figure 8:**
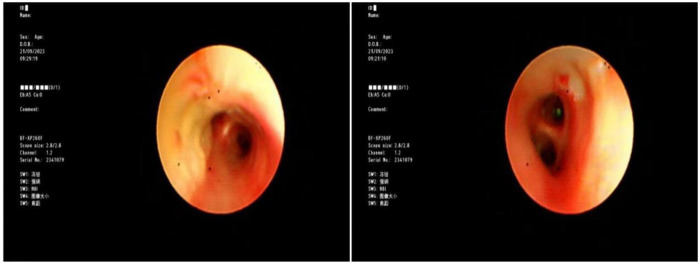
Bronchoscope (2023-09-21): complete epithelialization of the exposed cartilage.

**Figure 9:**
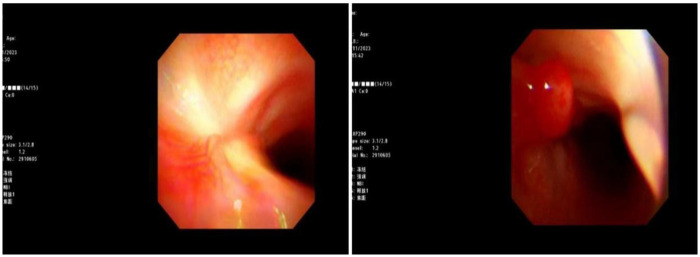
Bronchoscope (2023-11-09): show no cartilage exposure, see a little granulation hyperplasia.

**Figure 10:**
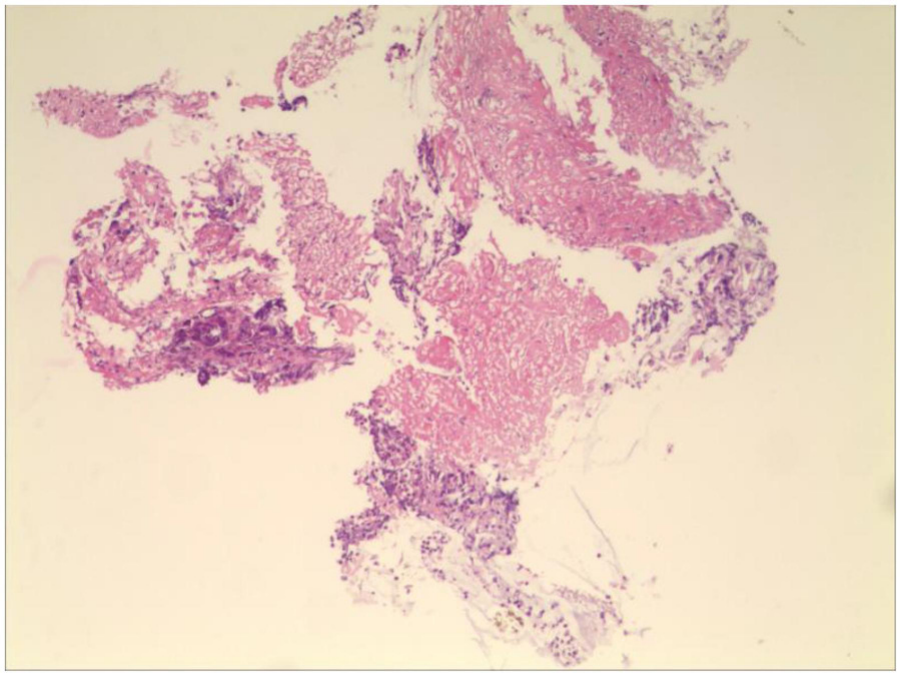
Pathology report: the main component is necrotic tissue, with inflammatory cells around, and no obvious granulomatous nodules are seen.

Blood gas and electrolytes: Sodium 129 mmol/L, others normal. After sodium supplementation, the re-examination was normal. Blood routine: White blood cells 10.52 × 10^9/L, hemoglobin 125 g/L, neutrophils 79.8%, platelet count 240 × 10^9/L, CRP 7.89 mg/L. Coagulation routine: D-dimer 2,940 ug/L. Biochemical profile: Lactate dehydrogenase 416.5 U/L. Ferritin normal. Cytokines: Interleukin-6: 30.43 pg/mL, Interleukin-10: 25.71 pg/mL, others normal. Negative for autoantibodies, ANA profile. Bronchoalveolar lavage fluid NGS: *M. pneumoniae* sequence count 58,005, coverage rate 97.2%. Negative for fungal quadruple, T-spot, Xpert. Lavage fluid cell morphology: Neutrophils 55%, Histiocytes 45%. Chest CT (2023-8-31): Detected a minor degree of inflammation in the lower lobe of the left lung (Figure 1); Chest MRI (2023-09-12): Inflammation in both lungs with bilateral pleural effusion, notably more pronounced on the left (Figure 2). Chest CT (2023-09-27): Patchy and strip-like high-density shadows in both lungs, particularly accentuated in the lower lobe of the left lung, with partial absorption compared to the previous scan (Figure 3). Post-discharge high-resolution chest CT (2023-10-26): Patchy and strip-like high-density shadows in both lungs, particularly accentuated in the lower lobe of the left lung, with partial absorption compared to the previous scan (Figure 4). Bronchoscopy (2023-09-04): Bronchitis, necrotizing tracheobronchitis (Figure 5). Bronchoscopy (2023-09-07): Bronchitis, necrotizing tracheobronchitis with evident cartilage exposure (Figure 6). Bronchoscopy (2023-09-14): Bronchitis and necrotizing tracheobronchitis with obvious cartilage exposure, along with softening of the left main bronchus post-infection (Figure 7). Bronchoscopy (2023-09-21): Bronchitis and necrotizing tracheobronchitis with a noticeable improvement in cartilage exposure (Figure 8). Bronchoscopy (2023-11-09): Bronchitis and necrotizing tracheobronchitis that had already undergone repair (Figure 9). Revealed predominantly necrotic tissue components surrounded by inflammatory cells, without the presence of apparent granulomatous nodules (Figure 10).

### Diagnosis

Severe and refractory MPP, necrotizing tracheobronchitis.

### Treatment course

Budesonide and a combination of ipratropium bromide were administered through nebulization to alleviate airway hyperresponsiveness and aid in cough expectoration. However, despite these interventions, the child continued to experience intermittent fever spikes, with a peak temperature of 39.3 °C. To address potential infection, azithromycin was introduced on September 3, 2023. The following day, on September 4, a bronchoscopy and lavage procedure were conducted, revealing substantial white, caseous, curd-like secretions in the primary airways and the left bronchus during the examination, accompanied by mucosal congestion, erosion, and a significant amount of viscous secretions in the airway lumen (Figure 6). Tests for T-spot, X-pert, and fungal infections yielded negative results. Analysis of the alveolar lavage fluid via Next-Generation Sequencing (NGS) revealed a *M. pneumoniae* sequence count of 58,005 with a coverage rate of 97.2%. Despite these efforts, the intermittent fever persisted, leading to an adjustment in treatment on September 5, 2023, with methylprednisolone dosage increased to 90 mg and a switch to cefoperazone-sulbactam as the antibiotic. By 2023-09-06, the child's temperature normalized. Following this, a series of three bronchoscopies were performed to monitor progress and response to treatment. On 2023-09-07, another bronchoscopy revealed a significant amount of white, caseous, curd-like secretions in the main airways and left main bronchus, along with mucosal congestion, erosion, and exposed cartilage rings (Figure 7). To assess the soft tissue and cartilage damage at the lesion site, an MRI was performed: inflammation of both lungs and pleural effusion on both sides, with the left lung being more pronounced (Figure 3). Biopsy of the lesion site showed predominant necrotic tissue components, with surrounding inflammatory cells, and no obvious granulomatous nodules were seen. On 2023-09-14, bronchoscopy revealed a significant amount of white, caseous, curd-like secretions in the main airways and left main bronchus, with mucosal congestion and erosion, exposed cartilage rings, and the left main bronchus collapsed by 1/2 during exhalation (Figure 8); On 2023-09-21, bronchoscopy showed a small amount of white necrotic mucosa attached to the wall of the main airways and left bronchus, with mucosal congestion and erosion, and exposed cartilage rings (improved compared to before) (Figure 9); The hormone was gradually tapered to oral administration, and the patient was discharged after improvement. After discharge, the patient was given a low dose of azithromycin, acetylcysteine orally, and budesonide and compound ipratropium bromide nebulization treatment. On 2023-11-09, a follow-up bronchoscopy showed a small amount of congestion and granulation of the main airway mucosa, no exposed cartilage rings, and the left bronchus mucosa had recovered well with no granulation (Figure 10). A follow-up chest CT showed significant absorption compared to before.

## Discuss

Acute pseudomembranous necrotizing tracheitis also known as acute fibrinous laryngotracheobronchitis (AFLTB), is an acute diffuse necrotizing inflammation of the mucous membranes of the larynx, trachea, and bronchi. This condition is frequently observed in young children, particularly during the winter and spring seasons, and is strongly linked to influenza outbreaks, demonstrating a severe clinical course and alarmingly high mortality rates. AFLTB is primarily caused by secondary bacterial infections consequent to viral infections, notably associated with the influenza virus. Gram-positive bacteria, notably Staphylococcus aureus, Streptococcus pneumoniae, and Haemophilus influenzae, are common culprits, with the combination of Staphylococcus aureus infection and influenza virus exhibiting the highest mortality rates. In addition to targeted anti-infective therapy, bronchoscopy may be required to clear pseudomembranes and necrotic tissues in the airway to alleviate respiratory distress (1). Most of the bacteria involved are Gram-positive, with prevalent strains such as Staphylococcus aureus, Streptococcus pneumoniae, and Haemophilus influenzae. Notably, the mortality rate is at its peak when Staphylococcus aureus infection coincides with influenza virus presence (2). Through a search using the keywords “AFLTB,” “Acute pseudomembranous necrotizing tracheitis,” and “child,” 10 articles were identified that provided relatively detailed reports on 18 cases (3–12) (Tables 1, 2). Among the patients with a clear etiology, the predominant pathogens were influenza A virus in combination with Staphylococcus aureus. One patient also had Mycoplasma pneumoniae infection, but this was concurrent with influenza A virus infection. Thirteen of the 18 cases exhibited dyspnea, and 4 cases presented with hoarseness. Only 4 cases had underlying diseases. Bronchoscopic findings showed necrotic tissue in all cases, with some patients exhibiting pseudomembranous necrosis. Nevertheless, in this instance, the child's alveolar lavage fluid NGS revealed solely *M. pneumoniae* infection, and there are currently no documented cases in the literature of AFLTB resulting from *M. pneumoniae* infection. Presently, Asian children exhibit a notably higher resistance rate to *M. pneumoniae* infection compared to those in European and American regions. In this scenario, the child's alveolar lavage fluid NGS indicated sole infection with *M. pneumoniae*, and as of now, there are no documented instances in the literature linking AFLTB to *M. pneumoniae* infection (13, 14). Infection by *M. pneumoniae* can cause direct harm to the epithelial cells at the infection site or damage due to the immune response it incites, leading to the dissolution and necrosis of epithelial cells, cilia destruction, and wall edema. These effects are evident in the alveolar lavage fluid NGS of the child's case, where only *M. pneumoniae* infection was detected. Presently, there are no documented instances in the literature linking AFLTB to *M. pneumoniae* infection (15). During bronchoscopic examination, AFLTB manifests as congested and edematous bronchial mucosa, proliferative granulation tissue, necrotic material overlaying the surface, purulent secretions within the lumen, lumen stenosis and obstruction, mucosal ulcer formation, and neoplasm-like alterations (14). The child was admitted to the hospital with fever and cough as the primary symptoms, facing recurrent high fever and a severe condition. By the 12th day of illness, bronchoscopy revealed copious white, dry, cheese-like curdled secretions in the main airway and left bronchus, alongside congested and ulcerated mucosa, as well as abundant viscous secretions in the lumen. Subsequent examinations on the 15th and 21st days showcased similar secretions in the airway and left main bronchus, with congested, ulcerated mucosa, visible exposed cartilage rings, and partial collapse of the left main bronchus during exhalation. The child's condition rapidly deteriorated, with evident deep ulcers reaching the cartilage layer observed during bronchoscopy. Biopsy results depicted predominant necrotic tissue components surrounded by inflammatory cells, without significant granulomatous nodules. In treating AFLTB, alongside active anti-infection therapy, clearing foreign bodies within the airway is crucial to maintain patency. Bronchoscopy stands as the sole effective and rapid method for removing these obstructions, playing a pivotal role in managing airway blockages caused by infections such as AFLTB. The removal of secretions and debris through bronchoscopy can significantly enhance respiratory function and improve overall outcomes (16). In the treatment of AFLTB, maintaining adequate fluid intake is crucial to prevent the formation of dry secretions, alongside performing local lavage during bronchoscopy. In severe cases, local medications can be utilized to stimulate mucosal repair and prevent airway blockage by secretions or shedding of necrotic material. During a subsequent bronchoscopic examination on the 28th day of the disease course, a small amount of white necrotic mucosa was found adhering to the walls of the main bronchus and left bronchus, along with congested and ulcerated mucosa. A small quantity of granulation tissue was observed at the exposed cartilage ring, indicating initial signs of repair at the necrotic sites. The child's clinical symptoms notably improved, leading to a smooth discharge. Over a month post-discharge, a follow-up bronchoscopy revealed repaired mucosa at severely affected sites, with some granulation tissue proliferation. During a half-year outpatient follow-up, no signs of dyspnea or activity intolerance were noted, indicating a favorable recovery trajectory.

**Table 1:** Clinical characteristics of 18 children with necrotizing bronchitis (3–12).

Case	Age	Gender	Clinical manifestation	Physical sign	Background disease	Pathogeny
1	12 years and 6 months	male	fever, cough	shortness of breath, difficulty in breathing, poor mental state	none	Influenza B, Staphylococcus aureus
2	1 year and 8 months	female	fever, cough	hoarseness, shortness of breath, difficulty in breathing, poor mental state	none	Staphylococcus aureus
3	2 years and 4 months	female	fever, cough	hoarseness, shortness of breath, difficulty in breathing, poor mental state	none	Influenza A, Staphylococcus aureus
4	8 years and 6 months	male	fever, cough, sore throat	hoarseness, shortness of breath, difficulty in breathing	none	not examined
5	7 years	female	fever, cough	hoarseness, shortness of breath, difficulty in breathing, poor mental state	none	Influenza A, Staphylococcus aureus
6	5 years	male	fever	shortness of breath, difficulty in breathing	Fanconi Syndrome	fungus
7	8 months	male	fever, cough	shortness of breath, difficulty in breathing, poor mental state	none	not reported
8	1 year and 11 months	female	fever, cough	difficulty in breathing	none	Influenza A, Staphylococcus aureus
9	9 years and 5 months	male	fever	difficulty in breathing	none	Influenza A, Staphylococcus aureus
10	8 years and 11 months	male	fever, cough	none	none	Influenza A, Staphylococcus aureus, Haemophilus influenzae
11	9 years and 10 months	male	fever, chest pain	none	none	Influenza A、Staphylococcus aureus
12	5 years and 2 months	male	fever	difficulty in breathing	none	Influenza A, Gram-positive cocci
13	9 years and 8 months	female	fever, cough	none	none	Influenza A, Adenovirus, Mycoplasma pneumoniae, Staphylococcus aureus
14	16 days	male	fever, cough	difficulty in breathing	none	Staphylococcus aureus
15	32 days	male	none	difficulty in breathing	none	Not examined
16	29 days	female	none	difficulty in breathing	pulmonary atresia with a ventricular septal defect, atrial septal defect, and PDA	Not examined
17	4 days	male	none	difficulty in breathing	Intrauterine Growth Restriction	Not examined
18	7 years	male	fever, cough, sore throat	hoarseness	none	Mycoplasma pneumoniae

**Table 2:** Instrument test results of 18 children with necrotizing bronchitis.

Case	Findings on bronchoscopy	Pathology	Imaging studies	Number of bronchoscopies	Clinical outcome
1	necrotic tissue, mucosal hyperemia	diffuse necrosis, exudate, accompanied by numerous neutrophils, pus cells, lymphocytes, and other inflammatory cells	Bilateral pneumonia, right-sided pneumothorax, right-sided chest wall subcutaneous emphysema, and bilateral pleural effusion	5	Improved
2	necrotic tissue, mucosal hyperemia	focal inflammatory necrosis	Pneumonia, more prominent in the left lower lobe, with right pleural reaction and a slightly hyperdense linear shadow in the upper segment of the trachea	1	Dead
3	necrotic tissue, mucosal hyperemia, Localized pallor, mucoid discharge	inflammatory necrotic tissue, numerous pus cells	Obscured main bronchus, right middle lobe pneumonia with atelectasis	Multiple times	Improved
4	necrotic tissue	inflammatory necrotic tissue	not reported	Multiple times	Improved
5	black necrosis, Mucosal hyperemia、erosion and bleeding, purulent discharge	not reported	Bilateral pneumonia	1	Dead
6	white necrosis, mucosal hyperemia	not reported	Bilateral pulmonary nodular infiltration in the lung bases	1	Dead
7	scab-like tissue	not reported	Hyperinflation of the left lung	1	Improved
8	pseudomembranous necrosis, mucosal hyperemia, edema and erosion	extensive necrosis with infiltration of lymphocytes, plasma cells, neutrophils and histiocytes	Bilateral pulmonary infection with stenosis and occlusion of the bronchi in the right middle and lower lobes and the basal segments of the left lower lobe	1∼3	Improved
9	pseudomembranous necrosis, mucosal hyperemia, edema, and erosion	fibrinoid necrosis with exudation and inflammatory cell infiltration	Mucus plugs are visible in the trachea and bronchi of the right middle and lower lobes	1∼3	Improved
10	white pseudomembranous necrosis, mucosal erosion, exudation and bleeding, yellow and purulent discharge	not reported	Consolidation and atelectasis are visible in the right middle lobe and left lower lobe of the lung	1	Improved
11	white pseudomembranous necrosis, mucosal erosion、hyperemia, edema andnecrosis, purulent discharge	fibrinoid necrosis with exudation and inflammatory cell infiltration	Multifocal pneumonia	1∼3	Improved
12	pseudomembranous necrosis, mucosal hyperemia, edema, and erosion, yellow and purulent discharge	not reported	Local consolidation pneumonia in the right lung	1∼3	Improved
13	white pseudomembranous necrosis, mucosal hyperemia, edema and necrosis, purulent discharge	not reported	Multiple pulmonary infections with focal consolidation	1	Improved
14	white pseudomembranous necrosis, mucosal edema,	inflammatory necrotic tissue	A few linear and patchy ill-defined shadows in the right lower lung	1	Dead
15	yellow fragmentary necrosis, mucosal petechial hemorrhage	acute inflammatory debris and necrotic epithelial tissue	No report	4	Improved
16	fragmentary necrosis, mucosal bleeding	late-stage necrosis with pseudomembrane formation in the mucosa and submucosa	No report	none	Dead
17	fragmentary necrosis, mucosal bleeding	late-stage necrosis with pseudomembrane formation in the mucosa and submucosa	No report	none	Dead
18	Mucosal necrosis and ulceration with prominent exposure of cartilage	Predominantly necrotic tissue components with surrounding inflammatory cells	Bilateral lungs show patchy and linear high-density shadows, with the left lower lobe being more prominent	5	Improved

AFLTB, though uncommon, progresses rapidly and carries a high mortality rate as shown in Table 1, out of the 18 cases, 6 cases had a final outcome of death. As of now, there are no documented cases attributing it to *M. pneumoniae* infection. Vigilance is crucial for clinical physicians. When faced with respiratory distress, consideration should extend beyond plastic bronchitis to include this condition. Studies suggest that recurrent bronchoscopic procedures might worsen tracheal mucosal edema, particularly challenging for children with respiratory and circulatory failure. Post-tracheostomy, enhancing airway care is advised. Tracheotomy may not alleviate obstruction above and below the incision, nor effectively clear lesions, especially in the subglottic region. For recurrent obstructions, emergency bronchoscopy and lavage alongside robust anti-infective measures, fluid support, and airway humidification are necessary. The pathogenesis of *M. pneumoniae* remains unclear, with direct damage by *M. pneumoniae* and abnormal host immune responses being key mechanisms, warranting intensified anti-inflammatory therapies. Even after relieving airway obstructions, patients often require multiple bronchoscopies and secretion clearance from the lesion site. Clinical practitioners should contemplate multiple bronchoscopies upon admission or lesion identification in children under bronchoscopic examination, with discharge following lesion site healing.
